# Developments in caloric measurements, materials, and devices at Calorics 2024

**DOI:** 10.1557/s43581-024-00119-w

**Published:** 2024-12-09

**Authors:** Xavier Moya, Mengfan Guo, Neil D. Mathur

**Affiliations:** Department of Materials Science, University of Cambridge, Cambridge, CB3 0FS UK

**Keywords:** energetic material, environmentally benign, phase transformation, thermodynamics, devices

## Abstract

**Abstract:**

Heating and cooling combined constitute the world’s largest form of end-use energy and the largest source of carbon emissions. It is therefore interesting to explore heat pumps based on caloric materials, which offer promising and environmentally friendly alternatives to gas combustion and vapor compression. The possibility of replacing these traditional methods of heating and cooling motivates the current research on caloric materials and devices. Here, we report the latest developments from the second biennial Calorics conference.

**Graphical abstract:**

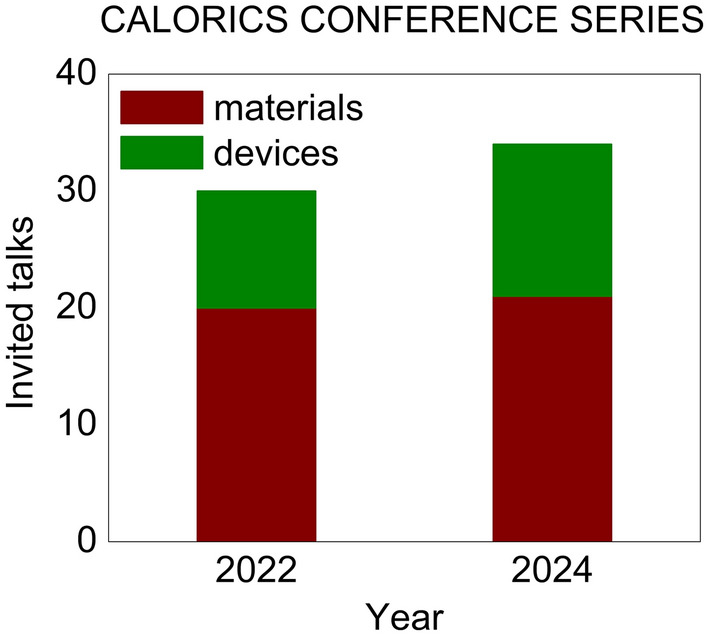

**Highlights:**

***Caloric materials offer environmentally friendly alternatives to traditional heating and cooling technologies, addressing global energy and carbon emission challenges. The latest developments on caloric measurements, materials and devices were presented at the second biennial Calorics conference in Cambridge (UK).***

**Discussion:**

How can caloric technologies be made more energy efficient to ensure they outperform traditional vapor-compression systems?

What are the most significant barriers to scaling caloric materials and devices from lab-scale prototypes to commercial applications?

Could integrating caloric technologies with renewable energy sources, such as solar or waste heat recovery, offer sustainable solutions for heating and cooling?

Calorics 2024 took place on 16–18 September 2024 at the Møller Institute in Cambridge (UK), lasting one day longer than the 2022 launch conference to reflect the upward trend in activity^1^ that aims to yield next-generation cooling and heating technologies.^2,3^ The event drew in seventy researchers, engineers, and innovators from fourteen countries (Fig. 1) to focus on caloric measurements, materials, and devices, while the 34 invited talks were divided not like this but rather into magnetocaloric, electrocaloric, elastocaloric, barocaloric, and multicaloric sessions. As with Calorics 2022, there were more materials talks than device talks (Fig. 2), but not one hundred times more, Andrej Kitanovski (University of Ljubljana, Slovenia) having informed the conference that there are one hundred times more materials papers than devices papers. The Calorics conference series is intended to push the boundaries of scientific understanding, foster collaboration, and promote applications, on this occasion lubricated by the first barocalorically cooled beer (Fig. 3).

**FIgure 1:**
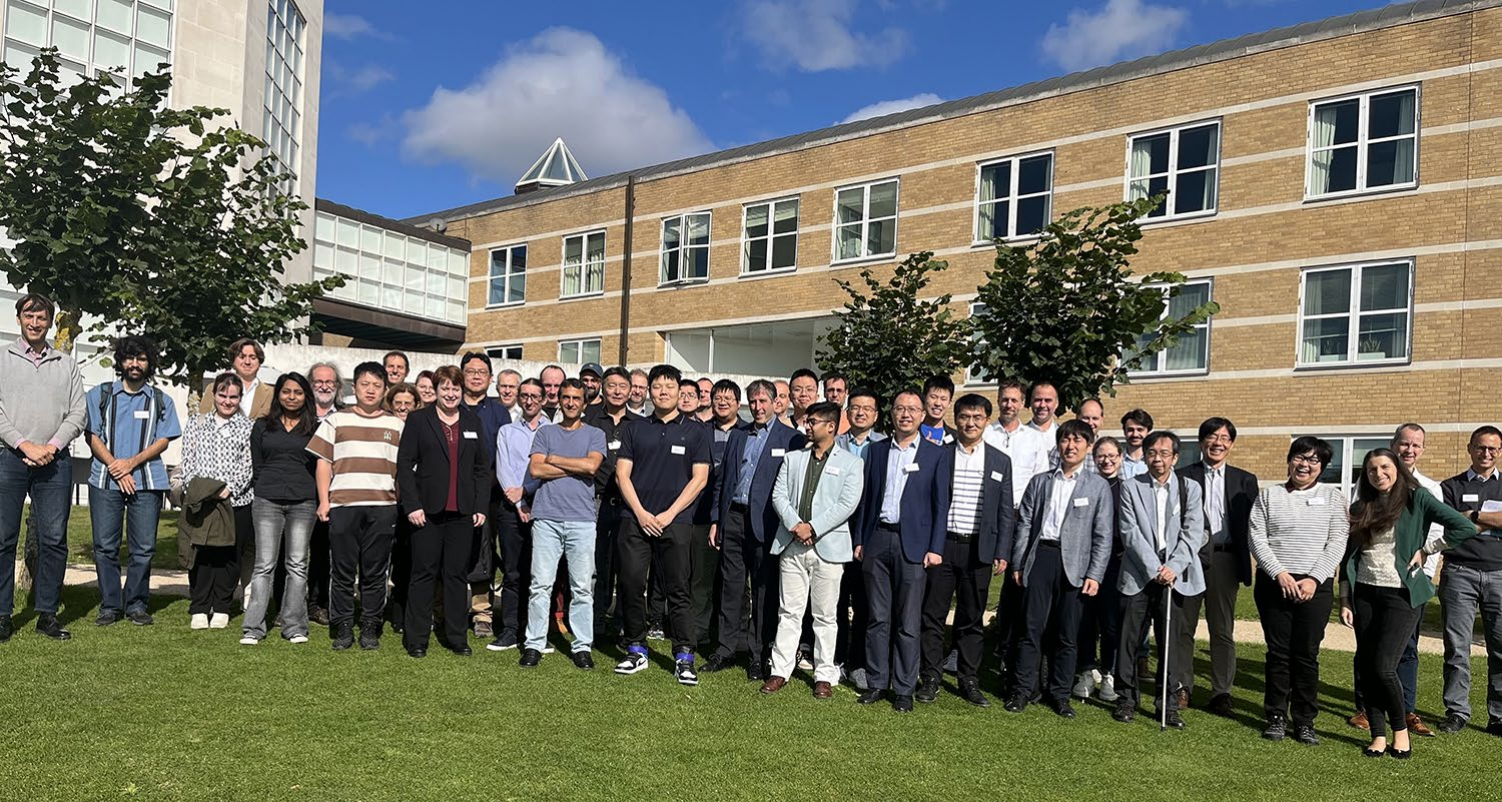
Participants at Calorics 2024 on the fields of Churchill College, Cambridge, UK. The Møller Institute in the background served as the conference venue.

**FIgure 2:**
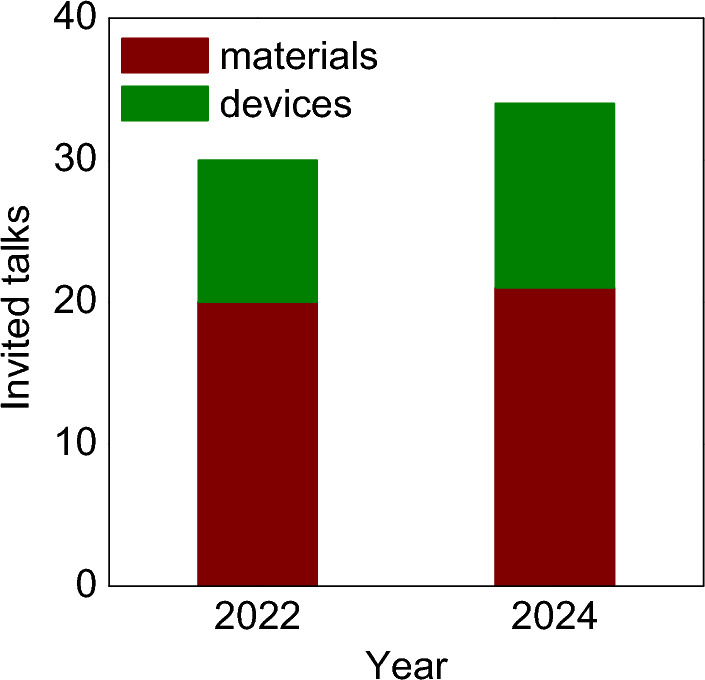
Invited talks on caloric materials and devices at the first two Calorics conferences.

**FIgure 3:**
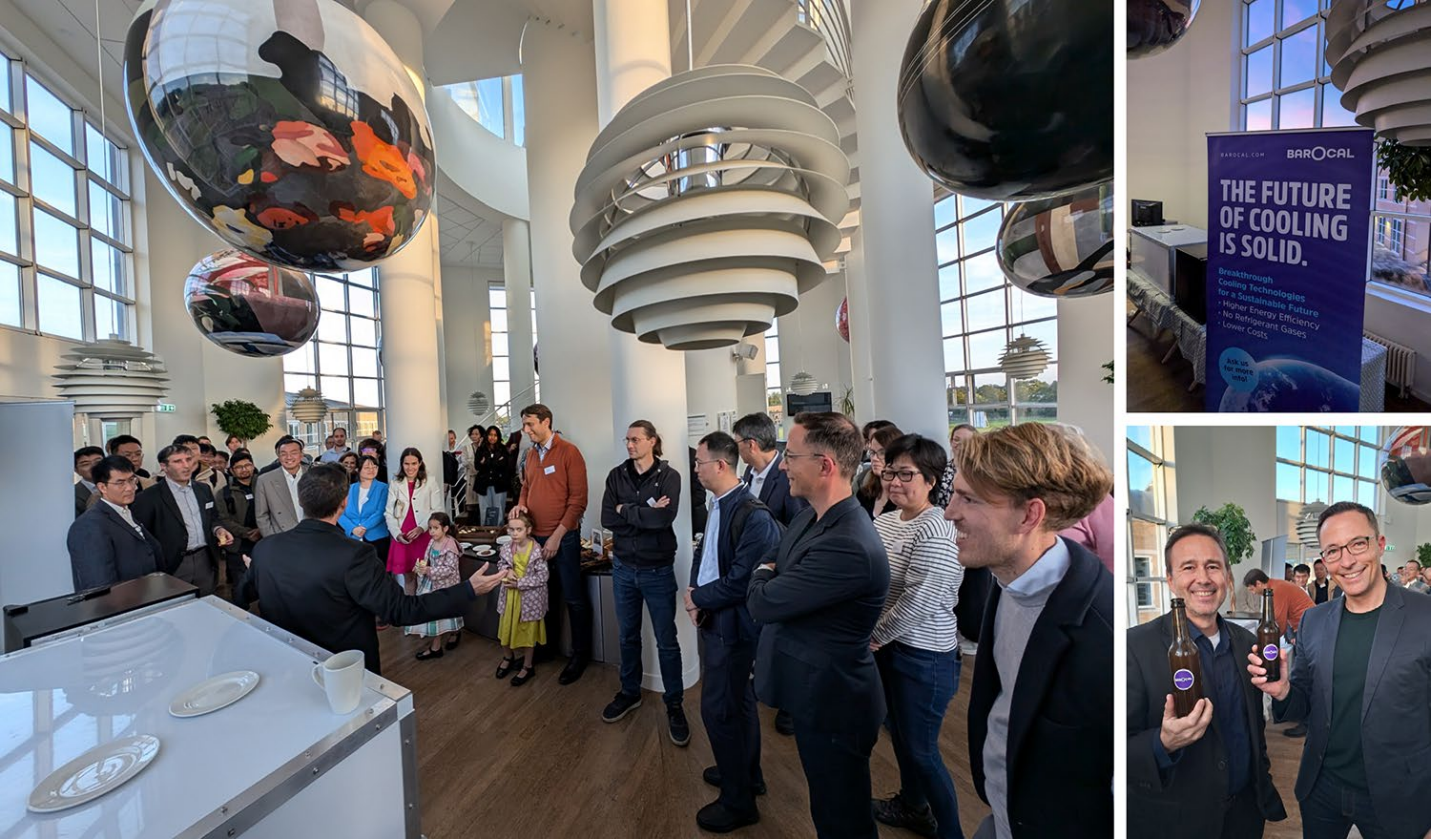
Barocal cooled a cabinet full of beer using a second-generation prototype that is the first barocaloric prototype ever to be unveiled.

## Magnetocaloric materials and devices

Andrej Kitanovski provided a comprehensive review of the challenges and opportunities in applying magnetocaloric materials for energy-efficient cooling and waste heat recovery, emphasizing their potential to significantly impact global decarbonization efforts. Jia-Yan Law (University of Seville, Spain) discussed the prospects of high-entropy alloys for magnetocaloric applications, highlighting the potential of property-directed exploration to optimize both mechanical and functional properties. Asaya Fujita (AIST, Japan) explored Fe-based magnetic refrigerants and introduced methods to optimize active magnetic regeneration performance using low magnetic fields, aiming to reduce the complexity of cascade stages in heat pump systems. Julie Slaughter (Ames National Laboratory, USA) presented strategies to reduce the cost and improve the specific power of both magnetocaloric and elastocaloric systems, aiming to achieve performance parity with traditional compressors. Finally, Franziska Scheibel (Technical University of Darmstadt, Germany) demonstrated how additive manufacturing can be used to enhance the design and mechanical stability of magnetocaloric and multicaloric materials, offering new possibilities for microstructural control and heat exchanger efficiency.

## Electrocaloric materials and devices

Kilian Bartholomé (Fraunhofer Institute, Germany) discussed a novel electrocaloric cooling system that leverages evaporation and condensation to enhance heat transfer, aiming for higher cooling power density. Sakyo Hirose presented research on antiferroelectric PbMg_0.5_W_0.5_O_3_ and its solid solutions, showing a temperature change of over 2 K for fields below 15 V µm^−1^, highlighting the potential of these materials for room-temperature cooling applications. Morgan Almanza (Université Paris-Saclay, France) explored the impact of dielectric losses on the efficiency of electrocaloric cycles, emphasizing the need for better loss characterization in polymers like poly(vinylidene fluoride–trifluoroethylene-chlorofluoroethylene) P(VDF-TrFE-CFE) to improve device performance. Barbara Malič (Jožef Stefan Institute, Slovenia) reported how doping influences PbMg_1/3_Nb_2/3_O_3_–PbTiO_3_. (PMN-PT) ceramics, demonstrating how lanthanum and manganese substituents modify dielectric properties and evaluating their radiation tolerance for use in harsh environments. Emmanuel Defay (LIST, Luxembourg) shared promising results on electrocaloric regenerators, achieving a temperature span of over 20 K near room temperature with several watts of cooling power. Qibing Pei (University of California Los Angeles, USA) and Yang Shen (Tsinghua University, China) both presented advances in electrocaloric polymers based on poly(vinylidene fluoride) (PVDF), focusing on optimizing molecular structures and inorganic fillers to boost performance in solid-state cooling devices.

Yang Bai (University of Science and Technology Beijing, China) discussed the critical role of phase transitions in enhancing the electrocaloric effect, emphasizing how different types of phase transition influence electrocaloric characteristics in ferroelectric materials. His talk highlighted materials, such as Pb-based ceramics, which can achieve both conventional and inverse electrocaloric effects. Rujun Ma (Nankai University, China) presented a novel approach using flexible ferroelectric polymer films for electrocaloric cooling, offering a portable, non-polluting solution for thermal management in wearable electronics. Xiaoshi Qian (Shanghai Jiao Tong University, China) introduced high-entropy materials based on PVDF polymers, which display enhanced electrocaloric effects at low fields, significantly extending the lifespan and efficiency of electrocaloric systems. Nikola Novak (Jožef Stefan Institute, Slovenia) explored barium zirconate-titanate ferroelectric solid solutions and how special points in the composition–temperature–electric field phase diagram enhance the electrocaloric effect.

## Elastocaloric materials and devices

Ichiro Takeuchi (University of Maryland, USA) presented advances in elastocaloric systems, particularly highlighting a design for a tube-bundle-based heat exchanger capable of switching between enhanced cooling capacity and a regenerator mode. He emphasized the importance of developing new materials, particularly Cu-based alloys, for improved performance. Mike Langan (Exergyn, Ireland) discussed the development of high-performance NiTi-based alloys for commercial applications, underscoring the potential of elastocaloric technology to surpass vapor-compression systems in both performance and environmental impact. Jaka Tušek (University of Ljubljana, Slovenia) shared results on fatigue-resistant elastocaloric devices, achieving a maximum temperature span of 30 K and calling for further improvements in the reduction of hysteresis losses. Gael Sebald (Centrale Lyon, France) explored the elastocaloric properties of natural rubber, presenting experimental systems based on natural rubber tubes and films, while also discussing challenges, such as thermal conductivity and deformation issues.

Ian Fisher (Stanford University, USA) presented research on the giant elastocaloric effect in the quadrupolar material TmVO_4_, highlighting its potential for cryogenic cooling and offering new insights into multipolar fluctuations. Andreas Rost (University of St Andrews, UK) shared findings from elastocaloric measurements on unconventional superconductor Sr_2_RuO_4_, emphasizing the ability of the method to reveal detailed thermodynamic properties and phase diagrams. Ken-ichi Uchida (National Institute for Materials Science, Japan) introduced an active thermal-imaging technique, using lock-in thermography to precisely measure elastocaloric effects, thus opening new possibilities for non-contact thermal measurements also in magnetocaloric and electrocaloric materials. Jingyuan Xu (Karlsruhe Institute of Technology, Germany) discussed the promise of superelastic shape memory alloy films for elastocaloric cooling, particularly due to the high surface-to-volume ratio, which enhances heat transfer efficiency in microcooling devices. Suxin Qian (Xi’an Jiaotong University, China) presented work on heat-driven elastocaloric cooling, showcasing a solar-thermal-driven prototype and emphasizing the potential of using low-grade heat sources to power elastocaloric systems. Lastly, Franziska Louia (University of Saarland, Germany) showcased a new method for comprehensive thermo-mechano-caloric characterization of elastocaloric materials, as well as the development of an elastocaloric cooling demonstrator that uses shape memory alloy wire bundles and air circulation.

## Barocaloric materials and devices

Pol Lloveras (Universitat Politècnica de Catalunya, Spain) presented finite-element modeling of barocaloric devices, demonstrating the potential of plastic crystals as refrigerants. His work highlighted how latent heat contributions deviate from typical cooling power curves, offering insights into optimizing barocaloric regenerator designs. Jarad Mason (Harvard University, USA) explored hydrocarbon order–disorder phase transitions in organic and metal–organic materials, discussing how structural and chemical factors contribute to large, reversible barocaloric effects at moderate pressures. Frederic Rendell-Bhatti (University of Glasgow, UK) shared new findings on the plastic crystal neopentyl glycol, using quasi-elastic neutron scattering to probe the molecular dynamics responsible for thermal hysteresis in these barocaloric materials. Gian Guzmán-Verri (University of Costa Rica, Costa Rica/University of Cambridge, UK) presented a Landau model of ferroelectric plastic crystals, capturing the interplay of electrical polarization and lattice strain in systems like quinuclidinium perrhenate. Yuichi Shimakawa (Kyoto University, Japan) discussed multiple caloric effects in transition metal oxides, particularly focusing on first-order phase transitions in perovskites such as NdCu_3_Fe_4_O_12_ and BiCu_3_Cr_4_O_12_, which exhibit coupled charge, spin, and lattice transitions. Lastly, Jing Wang (State Key Laboratory of Magnetism, China) presented a study of coupled caloric effects, showcasing barocaloric entropy changes enhanced by magnetic fields, with a focus on novel two-dimensional van der Waals materials.

## Multicaloric materials and devices

Lluís Mañosa (Universitat de Barcelona, Spain) presented research on the van der Waals ferromagnet Cr_2_Ge_2_Te_6_, exploring magnetocaloric and barocaloric effects, both separately and together. Significant magneto-structural coupling was found near the Curie temperature, where conventional magnetocaloric and inverse barocaloric effects were observed. Ab initio calculations supported the experimental findings, revealing the underlying electronic and magnetoelastic interactions responsible for the observed transitions. Enric Stern-Taulats (Universitat de Barcelona, Spain) then discussed multicaloric effects in poly(vinylidene fluoride–trifluoroethylene–chlorotrifluoroethylene) P(VDF-TrFE-CTFE) terpolymers, focusing on the synergistic application of electric field and mechanical stress. Franziska Scheibel also presented progress on a multicaloric device that operates under pulsed magnetic field and uniaxial stress.

## Outlook

Caloric technologies face both challenges and opportunities. The materials challenge is to improve caloric materials by reducing hysteresis, reducing fatigue, and increasing temperature span of operation, as these improvements are required for cooling/heating cycles that are efficient, repeatable, and useful, respectively. The device challenge is to modify prototype design in order to simplify existing complexity, achieve scale-up, overcome manufacturing issues, and increase cooling/heating power and energy efficiency in order to outperform traditional vapor-compression methods.

The materials opportunity could come in the form of advances in multifunctional materials, such as advanced electroactive polymers and hybrid organic–inorganic compounds, where improved caloric effects would imply improved device performance. The device opportunity could come from additive manufacturing for the fabrication of structures at multiple scales, e.g., for optimizing heat exchangers.

Beyond cooling and heating, caloric technologies could be applied to areas, such as waste heat recovery. Additionally, integrating caloric technologies into hybrid systems that utilize renewable energy presents a sustainable path forward, allowing for more efficient and environmentally friendly solutions.

As we plan Calorics 2026, we will reflect on the overwhelmingly positive feedback from the participants at Calorics 2024. Many highlighted the importance of the Calorics conference series in gathering the key players in the world under the roof of one small venue. Many praised the high quality of the presentations, as well as the extended networking opportunities that were facilitated by the many extended coffee breaks, a formal dinner on the second day, and an informal dinner on the final day. As we look to the future, we welcome the increased interest and significance of calorics in addressing the global energy challenges that we face.
